# Population Size, Sex and Purifying Selection: Comparative Genomics of Two Sister Taxa of the Wild Yeast *Saccharomyces paradoxus*

**DOI:** 10.1093/gbe/evaa141

**Published:** 2020-07-16

**Authors:** Vassiliki Koufopanou, Susan Lomas, Olga Pronina, Pedro Almeida, Jose Paulo Sampaio, Timothy Mousseau, Gianni Liti, Austin Burt

**Affiliations:** e1 Department of Life Sciences, Imperial College London, Ascot, Berks, United Kingdom; e2 Institute of Cell Biology and Genetic Engineering, NAS of Ukraine, Kyiv, Ukraine; e3 Department of Genetics, Evolution & Environment, University College London, United Kingdom; e4 UCIBIO, Departamento de Ciências da Vida, Faculdade de Ciências e Tecnologia, Universidade Nova de Lisboa, Portugal; e5 Department of Biological Sciences, University of South Carolina; e6 CNRS, INSERM, IRCAN, Universite Cote d’ Azur, Nice, France

**Keywords:** recombination, diversity, divergence, generation time, *ρ*, latitude

## Abstract

This study uses population genomic data to estimate demographic and selection parameters in two sister lineages of the wild yeast *Saccharomyces paradoxus* and compare their evolution. We first estimate nucleotide and recombinational diversities in each of the two lineages to infer their population size and frequency of sex and then analyze the rate of mutation accumulation since divergence from their inferred common ancestor to estimate the generation time and efficacy of selection. We find that one of the lineages has significantly higher silent nucleotide diversity and lower linkage disequilibrium, indicating a larger population with more frequent sexual generations. The same lineage also shows shorter generation time and higher efficacy of purifying selection, the latter consistent with the finding of larger population size and more frequent sex. Similar analyses are also performed on the ancestries of individual strains within lineages and we find significant differences between strains implying variation in rates of mitotic cell divisions. Our sample includes some strains originating in the Chernobyl nuclear-accident exclusion zone, which has been subjected to high levels of radiation for nearly 30 years now. We find no evidence, however, for increased rates of mutation. Finally, there is a positive correlation between rates of mutation accumulation and length of growing period, as measured by latitude of the place of origin of strains. Our study illustrates the power of genomic analyses in estimating population and life history parameters and testing predictions based on population genetic theory.

**Significance**

Simple population genetic theory predicts consistency in genomic data between patterns generated by processes in the short term and those in long term. For example, nucleotide diversity and linkage disequilibrium can be used to infer population size and rates of sex, which in turn are expected to affect the patterns of selection and rates of evolution. We analyze population genomic data from two phylogenetically independent lineages of the wild yeast *Saccharomyces paradoxus* and two outgroups and find significant differences in their population size and rates of sex. We also compare divergence from their common ancestor and find differences in rates of evolution and efficacy of selection consistent with the inferred differences in demography, thus illustrating the power of the approach.

## Introduction

Population genetic theory has identified some key demographic parameters that are expected to play a major role in the evolution of a species, including the population size, rates of sex and recombination, and generation time. Changes in these parameters are expected to affect various characteristics of a gene pool, and therefore population genomic data can be used to infer differences in these underlying parameters (Charlesworth and Charlesworth 2010). For example, differences in nucleotide diversity at silent sites can be used to infer differences in (effective) population size between two populations, and differences in the amount of linkage disequilibrium can be used to infer differences in rates of sex and recombination. Finally, divergence from a common ancestor is proportional to the number of generations since the divergence and therefore differences in divergence can be used to infer different generation times. Moreover, each of these demographic parameters is also expected to affect the process and consequences of natural selection, and these predictions can also be tested with population genomic data.

In this article, we analyze genomes from two sister lineages of the wild yeast *Saccharomyces paradoxus*, from Europe and Far East Asia. This close relative of *Saccharomyces cerevisiae* has now become a model organism for population and evolutionary genomics due to its nondomesticated status (Replansky et al. 2008; Boynton and Greig 2014; Marsit et al. 2017). In contrast to *S. cerevisiae*, it shows distinct population structure with several phylogenetically independent lineages (Koufopanou et al. 2006; Liti et al. 2009; Eberlein et al. 2019), which can be used as replicate or outgroup lineages to each other, allowing polarization of the direction of change within and between lineages. The European and Far East Asian lineages are the closest sister taxa, with ∼3% synonymous divergence (Bensasson et al. 2008), yet still are phylogenetically independent, showing compatible genealogies of unlinked loci and no shared polymorphisms (Koufopanou et al. 2006). A third lineage from the Americas with 10% synonymous divergence from the European, also known as *Saccharomyces cariocanus*, is also phylogenetically distinct, and shows further subdivisions within the lineage (Eberlein et al. 2019). We use *S. cariocanus* here as an outgroup to polarize changes during divergence of the European and Far East lineages from an inferred common ancestor. Previous work has shown that *S. paradoxus* undergoes infrequent sex and is highly inbred (Johnson et al. 2004; Tsai et al. 2008). There is also ample evidence for purifying selection in *S. paradoxus*, but little or no evidence for positive selection (Liti et al. 2009; Elyashiv et al. 2010; Vishnoi et al. 2011; Bergstrom et al. 2014; Koufopanou et al. 2015), making this a simpler system for testing predictions as selection is expected to prevail in only one direction.

Here, we use genomic data to compare the nucleotide and recombinational diversities in the European and Far East lineages and use them to estimate population size and the frequency of sex. Silent nucleotide diversity is generated in every cell division at a rate proportional to the rate of mutation per cell division, and remains relatively unselected. Similarly, recombinational diversity (i.e., diversity due to recombination events) is generated in every meiotic cell division, at a rate proportional to the rate of recombination per meiotic division. While coupled in obligately sexual organisms, sexual and asexual generations represent distinct processes in clonal organisms and their relative frequencies can be estimated from the ratio of these two measures (Tsai et al. 2008; Nieuwenhuis and James 2016).

We find that the European population is both larger and has more frequent sexual generations than the Far East population. We also analyze the divergence of the two lineages from their inferred common ancestor and find the European lineage to have had an apparent shorter generation time as manifested by increased accumulation of silent derived alleles (DAs) during its divergence. It also shows a higher efficacy of purifying selection against deleterious alleles, consistent with reduced effects of drift and interference between loci in a larger more sexual population. Finally, the analysis of divergence can also be performed within lineages, and we find that individuals within both populations differ from each other in the rates of mutation accumulation. In one population where data are available, these differences correlate well with the length of growing season as measured by geographic latitude of the place of origin of strains, consistent with individuals having more cell divisions in warmer climates. Included in the European lineage is a sample of strains isolated from the 1,986 Chernobyl nuclear-accident exclusion zone, thus allowing us to test for any effects due to high-radiation exposure for nearly 30 years (Moller and Mousseau 2016). We find no increased mutation however, in Chernobyl strains compared with other strains originating at similar latitude.

## Materials and Methods

### Genome Preparation and Assemblies

Genome libraries were prepared for each of 20 strains of *S. paradoxus* (standard genomic libraries for paired-end sequencing prepared by GATC; www.eurofinsgenomics.eu; last accessed July 21, 2020; see list of strains in supplementary appendix I, Supplementary Material online), which were then sequenced using Illumina technology (Illumina HiSeq 2000; paired-end sequencing, 100-bp-reads, expected insert size ∼500 bp; also by GATC). Illumina read files were also obtained for an additional eight strains of *S. paradoxus* available as part of the SGRP2 yeast sequencing project of the Sanger Centre (ftp://ftp.sanger.ac.uk/pub/users/dmc/yeast/SGRP2/input/strains/; last accessed July 21, 2020; Bergstrom et al. 2014), and for a third set of three strains, supplied by the Jose Paolo Sampaio group in Lisbon, Portugal (Illumina MiSeq; paired-end sequencing, ∼250-bp-reads, insert size ∼350–450 bp; facility in IGC/Lisbon; http://www.crem.fct.unl.pt/Yeast_Genomics_Lab/; last accessed July 21, 2020). All reads have been deposited to NCBI (Bioproject PRJNA 635028).

Illumina reads were mapped to a reference sequence of *S. paradoxus* (strain CBS432, for European strains, CBS8438 = N_44 for Far East strains and YPS138 for North American/*S. cariocanus* strains; pacBio sequences [Yue et al. 2017]; see also Scannell et al. 2011), using the Map-to-Reference function in Geneious (unless otherwise stated, default settings in Geneious [v. 9.1] were applied).

Paired-end reads were retained only if top-scoring for mapping position and with both ends mapping correctly (supplementary appendix II, Supplementary Material online gives the assembly statistics for all strains). For each strain, a majority consensus sequence was called from the mapped reads, that is, the allele with the highest absolute frequency in the reads was selected (Geneious setting: Threshold 0%). Only sites with at least 10× coverage were accepted (this resulted in >Q30 for 100% of bases retained and >Q40 for 99% of bases for the majority of strains).

### Aligning and Annotating Genomes

Consensus sequences for all chromosomes were concatenated to form a whole-genome sequence for each strain. Strains from Europe were aligned using the Geneious Multiple Align option. Far East strains were separately aligned, and the two alignments were then aligned to each other. Last, the *S. cariocanus* (strain YPS138) and the *S. cerevisiae* (strain S288C) genomes were added to the alignment. The entire alignment consists of 33 sequences, including the four reference sequences.

The alignment was annotated using the European *S. paradoxus* reference sequence, above (Yue et al. 2017); see also Scannell et al. (2011). To remove ambiguities of alignment due to gene duplications, all genes in the European reference were BLAST-ed against all others, and only those with no significant match were retained for analysis, and only genes located at the “core” of chromosomes (i.e., excluding subtelomeric regions, as defined by Yue et al. 2017). A total of 4,698 genes are included (out of 5,536 annotated in the Europe reference sequence), and only sites with no missing data in any of the strains in both populations and the two outgroup sequences are included in all the analyses.

### Ancestral and Derived Status of Alleles

Ancestral and derived states of alleles were assigned by comparison to outgroup sequences. For sites polymorphic in one population, the other population was used as outgroup, and for sites with fixed differences within populations, the North American sequence was used (sites where the status was uncertain were classified as “missing”). Codon positions and 0-, 2-, and 4-fold degenerate sites in the alignment were annotated based on the common ancestral sequence for both populations.

### Nucleotide Diversity, Recombination, and Frequency of Sex

Data were processed and analyzed using custom scripts in R and software in JMP (SAS Institute Inc.). Recombination was analyzed using the *LDhat* pairwise software with default values (https://github.com/auton1/LDhat; last accessed July 21, 2020; McVean et al. 2002). Recombinational diversity *ρ* was calculated for each chromosome separately (coding and noncoding sites). Two different runs of *LDhat* were performed and their average taken for each chromosome, weighted by length. To estimate the frequency of sex, *S*, that is, the number of sexual generations per mitotic generation, we used the estimates of *π* and *ρ* and the formula S = [(*r*/θ)(u/*r*)]/[(1+*F*)(1−*F*)] (derived from the equations *θ* = 4*N*_e_*u*/(1+*F*) and ρ = 4*N*_e_*rS*(1−*F*), where *F* is the inbreeding coefficient, *u, r* are the mutation and recombination rates, pbp, respectively; see also Tsai et al. 2008). *F* has previously been estimated as *F *=* *0.98 (Johnson et al. 2004). *θ* is estimated from nucleotide diversity (*π*) on 4-fold sites. Note that the (pbp) estimate for *ρ*, was derived by dividing the 4*N*_e_*r* estimate by the entire length of the reference sequence rather than the length of the filtered alignment used as input to *LDhat*, as this was thought to be more appropriate for deriving the estimate for the overall rate (i.e., 11,986,733 sites for EU and UK and 11,813,124 sites for FE). 

### Distribution of Fitness Effects

The distributions of fitness effects were estimated using the est_dfe software (http://www.homepages.ed.ac.uk/pkeightl; last accessed July 21, 2020; Schneider et al. 2011). The program first uses the category of “neutral” sites, here, the 4-fold sites, to estimate parameters for a demographic model that best describes the population and then uses it to make a prediction for neutral sites, against which the distribution of “selected” sites is tested. A mean estimate for deleterious effects, *4N*_e_*S*_d_, is given as a product of the effective population size, *N*_e_, and derived as a mean of an assumed gamma distribution of deleterious effects (mean of gamma is equal to *b/a=N*_e_*S*_d_, where *b, a* are the shape and scale parameters of the gamma distribution, respectively, and *S*_d_ is the estimate of deleterious effect); the proportions of sites at four different categories of severity of deleterious effect are also given. The program also allows testing for positive selection by comparing the likelihoods of models with varying proportions and magnitudes of advantageous mutations.

## Results

We analyze here an alignment of 23 *S. paradoxus* genomes from Europe, 11 of which are from the UK, and five from the 1986 Chernobyl nuclear-accident exclusion zone, thus having been subjected to a high-radiation environment for nearly 30 years (measuring up to 23 usv/h, ∼500 times the normal background radiation in uncontaminated control regions nearby; see supplementary appendix I, Supplementary Material online; Moller and Mousseau 2006; 2016). We also analyze eight strains from the Far-East Asian lineage, together with one sequence each from the North American lineage (also known as *S. cariocanus*), and from *S. cerevisiae* (see supplementary appendix I, Supplementary Material online for a list of strains and origins). Only coding regions are analyzed, except for the recombination analysis, which also included the noncoding regions (see Materials and Methods).

### Nucleotide Diversity and Population Size

**Fig. 1.**
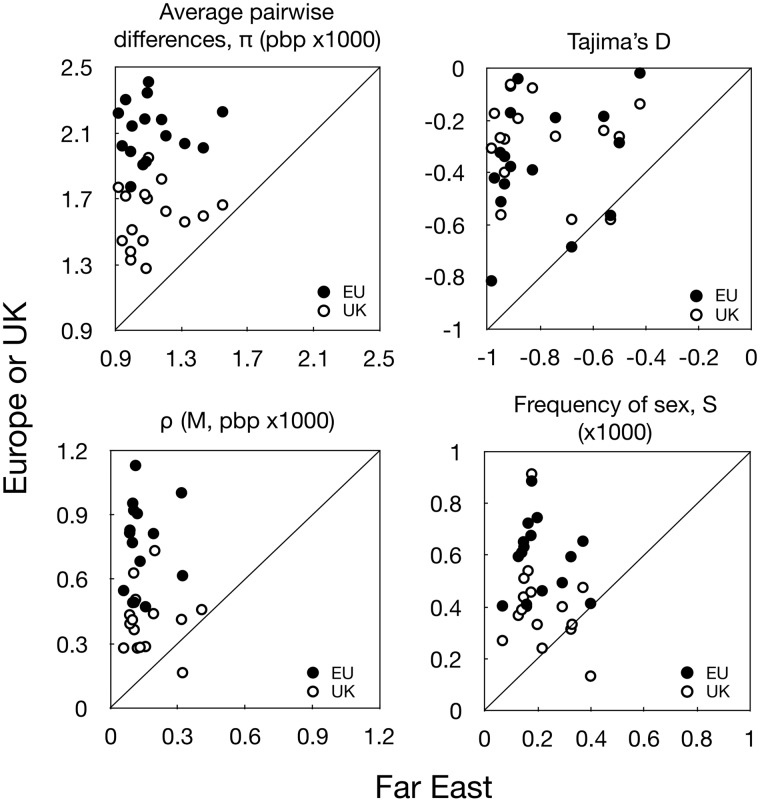
Population genetic parameters for the European and UK-only populations plotted against the Far East population. Points are for different chromosomes (*n* = 16). Top, left and right: Nucleotide diversity (average pairwise differences, *π*; pbp ×1,000) and Tajima’s *D* calculated for 4-fold degenerate sites. For diversity, all points are above the 1:1 line, showing higher diversity in both Europe and UK alone than in the Far East. Tajima’s *D* values are negative showing excess rare mutations in both populations, suggesting population expansion in both, with lower expansion in Europe. Bottom, left and right: Estimated recombination parameter (*ρ*) and frequency of sex (*S*) for European and UK-only populations, plotted against Far East. Again, points are above the 1:1 line, showing higher rates of recombination and frequency of sex, in both Europe and UK; note one point is omitted from the recombination plot (but included in all analyses), as it represents an outlier for recombination in Europe (but not in UK or FE).

To compare population sizes we calculated the average pairwise nucleotide diversity *π* at 4-fold degenerate sites across the entire genome. *π* is greater in Europe than Far East (*π *= 0.0021 vs. 0.0011, respectively, ∼1.9-fold higher in EU; paired *t*-test across 16 chromosomes *P* < 0.0001; table 1; fig. 1). This difference suggests a larger population size in Europe, and is possibly linked to the broader geographical sampling in Europe than Far East. To test this, we also compared *π* of UK isolates alone (which were all collected within a 10-km^2^ sampling area, similar to the Far East sampling; see supplementary appendix I, Supplementary Material online; Johnson et al. 2004) with that of Far East, and still find *π* larger in the UK (0.0016 vs. 0.0011, 1.45-fold higher, *P* < 0.0001).

**Table 1:** Nucleotide and Recombinational Diversity Estimates for the European, UK and Far East Populations

(A) Nucleotide Diversity (4-Fold Sites)						
	*N*	*N* Sites	***π***(pbp)	*P*	Tajima’s *D*	*P*
EU	23	882,812	0.0021	<0.0001	–0.34	<0.0001
UK	11	882,812	0.0016	<0.0001	–0.25	<0.0001
FE	8	882,812	0.0011	—	–0.83	—

(B) Recombinational Diversity								
	*N*	*N_Seg_*	***ρ***(M, pbp)	*P*	***ρ*/*θ*** (M)	*P*	*S*	*P*
EU	23	42,502	0.000815	<0.0001	0.388	<0.0001	0.000621	<0.0001
UK	11	24,505	0.000392	<0.0001	0.252	0.0008	0.000414	0.0008
FE	8	18,247	0.000133	—	0.122	—	0.000196	—

Note.—*N* is number of individuals sampled; *N_Seg_* is number of segregating sites; *S* is frequency of sex; *M* is Morgans; *P* is for difference from the Far East population.

To look for deviations reflecting potential changes in demography during the history of the two populations we also calculated Tajima’s *D*, which measures the standardized difference between *π*, above, and the sample size-corrected number of segregating sites, *θ*_w_. For neutral sites in equilibrium populations *D* should be close to zero (Tajima 1989). Negative values of *D* indicate excess of rare mutations characteristic of a star-like phylogeny in an expanding population, whereas positive values show excess of frequent polymorphisms, which may result from recent shrinkage (Haddrill et al. 2005). For 4-fold sites, *D* is negative for both Europe and Far East, suggesting population expansion in both, and more negative in Far East (4-fold sites only, chromosome averages weighted by length: *D* = –0.34 vs. –0.83, *P* < 0.0001; table 1; fig. 1). *D* is also negative for strains in the UK alone, different from Far East (–0.25 vs. –0.83, *P* < 0.0001), and not different from Europe (*P* = 0.18).

### Recombinational Diversity and the Frequency of Sex

To compare rates of recombination in the two lineages we calculated the recombinational diversity *ρ* for each chromosome using the computer software *LDhat* 2.2 (McVean et al. 2002). We find *ρ* greater in Europe than Far East (chromosome averages weighted by length: 0.000815 vs. 0.000133 M/bp, 6.13-fold higher in EU, paired *t*-test across 16 chromosomes, *P* < 0.0001; table 1; fig. 1). The expected effect of the broader geographic range in Europe is not clear in this case, as on one hand Europe may encompass a larger population, and therefore larger *N*_e_. On the other hand, the broader geographic range may entail geographic differentiation in at least some parts of the range, which will in turn create correlations between alleles, that is, linkage disequilibrium, thereby decreasing *ρ*. Previous analyses show both extensive recombination and mixing of strains across Europe (indicated by complete incompatibility of gene genealogies) and geographic differentiation in allele frequencies among different regions in Europe (Koufopanou et al. 2006). When data are analyzed for the UK alone, *ρ* is lower than in Europe, but still larger than Far East (0.000392 vs. 0.000133 M/bp, 2.95-fold larger than FE; *P* < 0.0001; table 1; fig. 1).

To estimate the frequency of sex, *S* (i.e., number of sexual generations per mitotic generation) in the two populations we used the estimates of *θ* and *ρ* and the method of Tsai et al. (2008) (see Materials and Methods). We find *S* three times larger in Europe than Far East (0.000621 vs. 0.000196, 3.17-fold larger than FE; *P* < 0.0001; table 1; fig. 1). These values correspond to an average of one sexual generation every 1,610 or 5,102 mitotic generations for Europe and Far East, respectively. When comparing with the UK alone, the estimated frequency of sex is about twice that of the Far East (0.000414 vs. 0.000196, 2.11-fold higher; *P* = 0.0008). Previous modeling has shown that the ratio *ρ*/*θ* can also be affected by changes in population size, becoming smaller after a bottleneck (Haddrill et al. 2005; O'Loughlin et al. 2016). Assuming the effect of population expansion on this ratio is in the opposite direction, that is, *ρ*/*θ* becomes larger following population expansion, our estimates for the frequency of sex may be an overestimate of the true value, for both populations.

### Divergence from the Common Ancestor

#### Generation Time

**Fig. 2.**
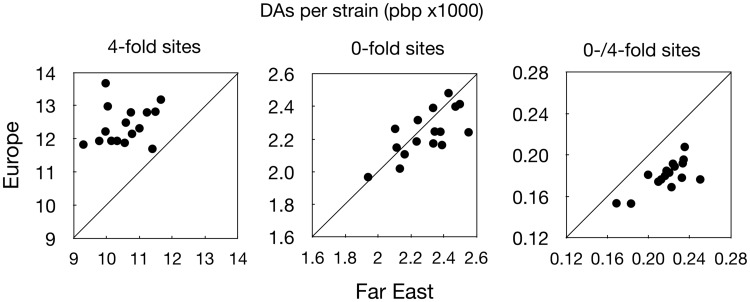
Divergence of European and Far East populations from their inferred common ancestor for 0- and 4-fold degenerate sites and the 0-/4-fold ratios (number of derived alleles [DAs] per strain, averaged across strains; points are for different chromosomes). Note ratios for Europe are below the 1:1 line, indicating stronger purifying selection in Europe.

Like most microbes, yeast can have greatly varying replication rates depending on environmental conditions, and in addition can also have a dormant ascus phase. It is conceivable therefore that the two lineages might differ greatly in the number of generations in the time since their common ancestor. To test for such differences we calculated the divergence at 4-fold sites from the inferred common ancestor of the two lineages (this includes both fixed and polymorphic differences, i.e., DAs; see Materials and Methods). On average, European isolates have accumulated more 4-fold DAs since their common ancestor than Far East isolates (0.0124 vs. 0.0104 DAs per base pair (pbp), per strain, 1.19-fold higher in EU; paired *t*-test across 16 chromosomes, *P* < 0.0001; table 2; fig. 2). This implies that the mutation rate per unit time has been higher in the European lineage. If we assume most mutations result from mitotic cell divisions, and that the mutation rate per cell division is the same in the two lineages, this implies that the European lineage has been slightly more mitotically active or has had a higher replication rate than the Far East lineage. Note we are not repeating the analysis for the UK-alone isolates as any differences between subpopulations will be due to very short-term divergence within lineages and relatively small compared with the longer-term divergence between the two lineages (see also comparison between fixed vs. polymorphic sites, below).

**Table 2:** Divergence of European and Far East Populations from Inferred Common Ancestor

		4-Fold		0-Fold		0-/4-Fold		
	*N*	*N* Sites	DAs/Strain (pbp)	*N* Sites	DAs/Strain (pbp)	All Sites	Fixed	Polymorphic
EU	23	882,812	0.01236	4,468,373	0.00228	0.18431	0.17765	0.21483
FE	8	882,812	0.01044	4,468,373	0.00232	0.22174	0.2157	0.2787
P	—	—	<0.0001	—	0.08	<0.0001	<0.0001	<0.0001

Note.—Numbers of derived alleles (DAs); *P* is for difference from the Far East population.

#### Purifying Selection

As the European population is both larger and more sexual, we would also expect it to be under more effective purifying selection. To test this hypothesis we compared the ratio of 0–4-fold DAs. The divergence of each of the two lineages from their common ancestor at 0-fold sites is slightly lower in Europe than Far East (0.00228 vs. 0.00232 DAs per strain, pbp; *P* = 0.08; table 2; fig. 2) and therefore the ratio of divergences at 0-/4-fold sites is also lower in Europe (0.184 vs. 0.222, 1.21-fold lower in EU than FE, *P* < 0.0001; table 2; fig. 2), indicating more effective purifying selection in Europe, as expected.

We can further partition the sites into those that are polymorphic in a population, that is, have both derived and ancestral alleles segregating, and those fixed for the DA. The 0/4-fold ratios are higher in the polymorphic sites, as would be expected for recent mutations with less opportunity to have been selected against, compared with fixed sites where selection would have been cumulative over more generations (polymorphic: EU vs. FE: 0.215 vs. 0.279, respectively, 0.77-fold lower in EU; *P* < 0.0001; fixed: 0.178 vs. 0.216, 0.82-fold lower in EU; *P* < 0.0001; fixed vs. polymorphic: *P* < 0.0001 for both EU and FE; 1.21- and 1.29-fold lower, respectively; table 2). Again, lower ratios in Europe than Far East indicate higher efficacy of selection in Europe. This difference is also seen in a lower *π*0/*π*4 ratio in European yeast (0.24 vs. 0.28 for Europe and UK, vs. Far East, respectively; *P* < 0.0001).

### The Distribution of Fitness Effects

#### Purifying Selection

**Fig. 3.**
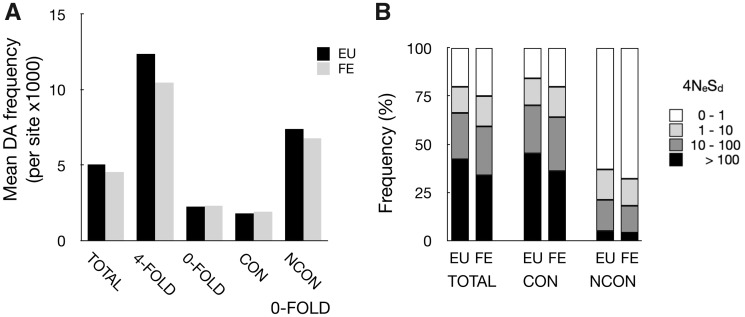
(*A*) Mean frequency of derived alleles (DAs) per site at different categories of sites in Europe and Far East. (*B*) Proportions of sites in different categories of severity of deleterious effect (4*N*_e_*S*_d_) in Europe and FE; all sites (TOTAL); outgroup-aa. Conserved (CON); and nonconserved (NCON).

To analyze purifying selection in more detail we compared the distribution of fitness effects in the two populations. These can be estimated from the distributions of DAs among two different categories of sites, one that is assumed to be neutrally evolving and one that is under selection (using the *est_dfe* software; Schneider et al. 2011). For both populations, the demographic history is best described with a model that includes at least one change in size during their history (*P* < 0.0001 for both populations). For both populations, the change is an increase, consistent with the Tajima’s *D* result above. Considering purifying selection, the mean estimate for deleterious effects, *N*_e_*S*_d_, is larger in Europe than in Far East (347.2 vs. 207.2, respectively, 1.67-fold higher in EU; table 3), indicating stronger selection against deleterious effects in Europe, consistent with the predictions above. This difference is very similar in magnitude to the difference in *N*_e_ as estimated from nucleotide diversity, *π* (tables 1 and 3; fig. 3).

**Table 3:** Parameters Estimated by the *est_dfe* Model

	*N* Sites						
	0-Fold	4-Fold	*n*	*b*	*E*_s_	4*N*_e_*S*_d_	Log *L*
EUROPE							
Total	4,468,373	882,812	2	0.23	–3.35	347.24	–159,258
CON^a^	4,085,917	882,812	2	0.27	–3.3	342.17	–123,367
NCON	379,909	882,812	2	0.1	–0.16	16.39	–33,482
CON + NCON^b^	—	—	—	—	—	—	–156,849
FAR EAST							
Total	4,468,373	882,812	2	0.22	–1.57	207.18	–107,724
CON	4,085,917	882,812	2	0.26	–1.51	198.34	–84,473
NCON	379,909	882,812	2	0.09	–0.11	14.21	–21,689
CON + NCON	—	—	—	—	—	—	–106,162

Note.—Only deleterious effects were estimated (assumed *gamma* distributed, where *b* and *E*_s_ are the shape and scale parameters of the distribution and 4*N*_e_*S*_d_ is the mean deleterious effect; *n* is the number of parameters estimated by the model).

a CON, NCON are for sites that are Conserved or Nonconserved at the amino-acid level between the *Saccharomyces cariocanus* and *Saccharomyces cerevisiae* outgroup sequences, respectively; note that both Conserved and Nonconserved 0-fold site distributions were tested against the total 4-fold site distributions, to maintain the same standard for all comparisons.

b Likelihood ratio tests for Conserved and Nonconserved categories, combined versus separate; Likelihood: Total – (CON + NCON): –159,258 – (–156,849) = –2,409; df = 4 – 2 = 2, and –107,724 – (–106,162) = –1,561, df =4 – 2 = 2, for Europe and Far East, respectively; *P* < 0.0001 for both.

#### Positive Selection

Our data again confirm that there is little or no evidence for positive selection overall, as models with nonzero proportions of substitutions due to positive selection are not significantly more likely than those with zero positive effects in either population (same models as above, but setting either the proportions of loci with positive, or magnitude of effects, to nonzero and comparing the Likelihoods to models where either of the two parameters are fixed to zero values). Sites that differ between closely related species might be more likely to show evidence for positive selection, as they would on average be more tolerant to change. To test this, we analyzed separately 0-fold sites in codons that code for the same amino acid in two outgroup species (*S. cariocanus*, the North American lineage, vs. *S. cerevisiae*) and sites that code for different amino acids in the two outgroups (Koufopanou et al. 2015). About 8.5% of 0-fold sites are “outgroup-non-conserved.” As expected, these sites show weaker purifying selection in both Europe and Far East (>10 times lower *N*_e_*S*_d_, for both populations; Likelihood ratio tests, *P* < 0.0001; table 3; fig. 3). However, even in this group of sites there is no significant evidence for adaptive mutations.

### Variation between Individuals within Populations

Having found differences between the two populations in the rate of accumulation of DAs, we now turn to look for similar differences among strains in the same population. In Europe there are 28,480 polymorphic sites and the number of DAs in a strain ranges between 6,921 and 6,468, a difference of 453 nucleotides. For Far East, there are 11,419 polymorphic sites and the number of DAs ranges from 3,556 to 3,212, a difference of 344 nucleotides. To test whether these differences are significant, we randomized the DAs among individual genomes (while maintaining the frequencies of alleles within sites unchanged) and compared the variance in the number of DAs among the observed genomes to the distribution of variances obtained among the randomized genomes. For both populations, we find that the variance of observed genomes is larger than the variances obtained from 1,000 randomizations, indicating that individuals differ significantly from each other in numbers of DAs (*P* < 0.001), or in other words there is positive linkage disequilibrium for DAs among individuals.

**Fig. 4.**
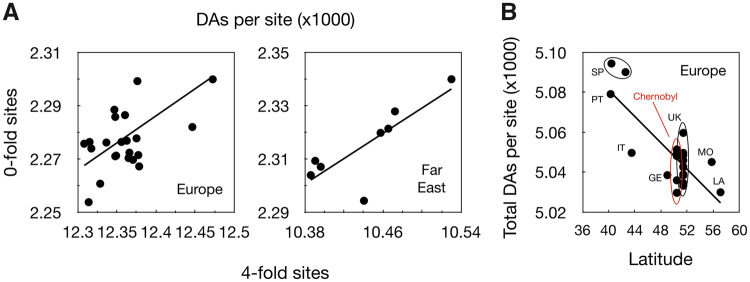
(*A*) Correlation across strains between the number of derived alleles (DAs) at 0- versus 4-fold sites (Europe: *r* = 0.56, *P* = 0.005; Far East: *r* = 0.81, *P* = 0.02). (*B*) Correlation with Latitude of place of origin (total DAs; European strains only; *r* = 0.80, *P* < 0.0001). Points are for different strains; five strains from Chernobyl and 11 strains from the UK and two from Spain are circled; SP Spain; PT Portugal; IT Italy; GE Germany; MO Moscow, Russia; LA Latvia.

In both populations, there is also a significant correlation between the number of DAs at 0- and 4-fold sites across individuals (*r* = 0.56, *P* = 0.005 and *r* = 0.81, *P* = 0.02, for EU and FE, respectively; fig. 4*A*), again showing differences among individuals in the numbers of DAs. There was no significant variation, however, among individuals in the ratio of 0-/4-fold DAs (1,000 randomizations, EU and FE *P* > 0.2). The Chernobyl strains might have been expected to show increased rates of mutation, due to their high-radiation exposure, however, they do not seem to differ from strains originating outside Chernobyl (*P* > 0.50; see also fig. 4*B*).

Last, we also looked for a possible effect of length of growing season, using latitude as a proxy. We hypothesized that lower latitudes would allow longer growing seasons, leading to more cell divisions per year in strains from the south. We could only apply this test for the European population, where we have samples from a range of latitudes, while all Far East strains are from about the same latitude. We find a significant correlation in the expected direction, with strains from southern Europe having more DAs than those from the north (*r* = 0.80, *P* < 0.0001; fig. 4*B*). This correlation is also consistent across chromosomes (negative correlation in 13 out of 16 chromosomes; *P* = 0.01, one-tailed sign-test).

## Discussion

We first describe the populations in the two lineages of *S. paradoxus* in terms of genetic diversity measured by pairwise differences among individuals and recombinational diversities, measured by the amount of linkage disequilibrium. Both are a function of population size, and we find a difference in both, implying the two populations differ in (effective) population size. We also find a difference in the ratio of the two diversities indicating a difference in the frequency of sex.

For these calculations, we have implicitly assumed that both rates of mutation and recombination, *u* and *r*, have remained relatively unchanged during the divergence of the lineages analyzed, as they are such close relatives, and slower to evolve than other more environment-dependent parameters, such as generation time, mating system and population size. As we have also found some evidence for population expansion during the history of the two populations, we cannot rule out the possibility that part of the apparent difference in frequency of sex may be due to some difference in demographic history of the two populations rather than a true difference in rates of sex and recombination. Differences in population size between close relatives are well documented, together with discussions of the evolutionary consequences of such differences (reviewed by Ellegren and Galtier 2016). Less well known, however, are differences in rates of sex or recombination, as most studies are done on obligate sexual organisms.

We then look at the accumulation of DAs in the two lineages since their divergence from common ancestor. The rate of accumulation of silent DAs is proportional to the rate of cell divisions since the common ancestor and can thus be used to estimate the relative generation times in the two lineages (see also Gibson et al. 2018 using similar methods in estimating generation times in bacterial populations). We find a shorter generation time in Europe, implying this lineage might have been mitotically more active than the Far East lineage. The ratio of expressed to silent mutations accumulated in the two lineages indicates the efficacy of selection during divergence of the two lineages, and we find a lower ratio in Europe indicating stronger purifying selection as would be predicted by its larger population size acting to reduce the power of drift, and higher frequency of sex reducing interference between linked loci (Charlesworth and Charlesworth 2010). As expected, the ratio is lower for mutations that have become fixed in the two populations through multiple rounds of selection and higher for mutations that are still segregating within populations. Consistent with previous studies in yeast, there is no evidence for positive selection even when only analyzing sites that have changed in outgroup species, and are likely to be relatively unconstrained (Liti et al. 2009; Gossmann et al. 2012; Koufopanou et al. 2015).

The finding of significant differences in purifying selection between the two populations suggests that the different attributes of the two, that is, larger population size and more frequent sex, have been in existence for a reasonable fraction of time since their divergence from the common ancestor for their effects to be measurable. Again, it could be that some of the difference in the strength of selection may reflect a difference in demographic rather than selective history in the two populations. Simulation studies show, however, that population expansion may result in more relaxed selection and higher frequency of deleterious mutations (Gazave et al. 2013), which is opposite to our finding of stronger selection in the larger European population.

Differences in strength of purifying selection are known between species of *Drosophila* varying in population size (Andolfatto et al. 2011) or between regions of the genome varying in rates of recombination (Haddrill et al. 2007; Campos et al. 2014; Singh et al. 2014). Reduced purifying selection in asexual compared with sexual lineages was shown in stick insects and snails (Bast et al. 2018; Sharbrough et al. 2018). In plants, more relaxed purifying selection was found in selfed species, compared with outcrossed (Chen et al. 2017). Chen et al. (2017) have also summarized the efficacy of purifying selection within species in a variety of animal and plant taxa, as measured by the *π*0/*π*4 ratio, and our values for yeast (0.24, 0.28 for Europe and Far East, respectively) fall well within the range observed in the survey (0.08–0.40).

We also used similar analyses to compare the numbers of DAs accumulated in the ancestries of individuals within the European and Far East populations and we find differences within both populations, showing the results are repeatable across populations. Assuming no differences in rates of mutation between strains, this may suggest variation in the rates of mitotic cell divisions, with some individuals having had more active ancestries than others. Individuals differ in both silent and expressed mutations, and there is little or no consistent evidence that strains have experienced different efficacies of purifying selection. This might have occurred if for example the local populations within these two lineages had been of significantly different sizes, or rates of sex, etc. More extensive sampling of local populations will help clarify further the causes for these differences among individuals.

As some of our strains originated in the Chernobyl region we tested for a potential effect of increased radiation on the number of DAs and found no difference. Perhaps these strains are recent immigrants to the region from other uncontaminated areas nearby, or have been dormant for a very long time and relative unaffected from radiation. Similar lack of evidence for increased genetic diversity or rates of substitution in regions of higher exposure to radiation in the Chernobyl exclusion zone (compared with nonirradiated controls nearby) was found in the plant–pathogenic fungus *Microbotryum* and the freshwater crustacean *Asellus* (Aguileta et al. 2016; Fuller et al. 2019). More extensive sampling of the region will be needed for a more definite answer. This is one of the first studies to provide whole-genome estimates of rates of new mutations in a eukaryote originating in the Chernobyl exclusion zone.

A possible explanation for the differences in DAs among individuals might be the length of growing season in their local environments, as our data suggest that individuals from the South, which are more likely to be experiencing warmer periods for longer, have been accumulating more DAs than those from the colder climates. Faster rates of molecular evolution or nucleotide diversity have been associated with lower latitude and/or warmer climates in a number of organisms (Davies et al. 2004; Gillooly et al. 2005; Machado et al. 2016; Barrera-Redondo et al. 2018), but our study is among the first to show clear evidence for an effect of latitude on rates of mutation accumulation. More evidence from different independent lineages is desirable to further confirm the robustness and repeatability of this result.

To conclude, our analyses illustrate the power of using genomic data to estimate population and evolutionary parameters in nature and using them to test predictions derived from population genetic theory. We have compared two close but phylogenetically independent lineages of the wild yeast *S. paradoxus* and have found patterns as expected by theory. More comparisons across several pairs of lineages of the same and other organisms will provide further tests and confirm the repeatability of observed patterns. Microbes, including pathogens, are particularly suited for such analyses and the increasing availability of population genomic data will be instrumental in providing critical information about their life histories.

## Supplementary Material

evaa141_Supplementary_Data
