# Supplementary material for: Population Size, Sex and Purifying Selection: Comparative Genomics of Two Sister Taxa of the Wild Yeast Saccharomyces paradoxus
Source: Genome Biol Evol. 2020 Jul 16;12(9):1636–45. doi: 10.1093/gbe/evaa141 (PMC7533043; doi:10.1093/gbe/evaa141)
Supplement: evaa141_Supplementary_Data [file evaa141_supplementary_data.docx]

Appendix I. List of strains

| Strain/CBS NO. | Latitude | Location* | Radiation ** | Reference |
| --- | --- | --- | --- | --- |
| EUROPE |  |  |  |  |
| CBS432 | 55.76 | Moscow, Russia |  | Yue et al. 2017 |
| CH1 | 50.42 | Chernobyl, Red Forest, Kiyv, Ukraine | 17 usv/hr | this study |
| CH2 | 50.42 | Chernobyl, Red Forest, Kiyv, Ukraine | 23 usv/hr | this study |
| CH4 | 50.42 | Chernobyl, Red Forest, Kiyv, Ukraine | 9.1 Mr/hr | this study |
| CH7 | 50.42 | Chernobyl, Krash, Kiyv, Ukraine | 0.06 usv/hr | this study |
| CH10 | 50.42 | Chernobyl, Parishev, Kiyv, Ukraine | 0.034Mr/hr | this study |
| SIG1 | 57.11 | Sigulda, Latvia |  | this study |
| DBVPG4650 | 43.51 | Marche, Italy |  | this study |
| CECT10176 | 40.42 | Spain |  | this study |
| T76_6 | 51.41 | Silwood Park, Ascot, UK |  | this study |
| Z3 | 51.41 | Silwood Park, Ascot, UK |  | this study |
| T8_1 | 51.41 | Silwood Park, Ascot, UK |  | this study |
| Q59_1 | 51.41 | Silwood Park, Ascot, UK |  | Bergstrom et al. 2014 |
| Q95_3 | 51.41 | Silwood Park, Ascot, UK |  | Bergstrom et al. 2014 |
| S36_7 | 51.41 | Silwood Park, Ascot, UK |  | Bergstrom et al. 2014 |
| Y7 | 51.41 | Silwood Park, Ascot, UK |  | Bergstrom et al. 2014 |
| Y8_5 | 51.41 | Silwood Park, Ascot, UK |  | Bergstrom et al. 2014 |
| Y9_6 | 51.41 | Silwood Park, Ascot, UK |  | Bergstrom et al. 2014 |
| Z1 | 51.41 | Silwood Park, Ascot, UK |  | Bergstrom et al. 2014 |
| Z1_1 | 51.41 | Silwood Park, Ascot, UK |  | Bergstrom et al. 2014 |
| ZP600 | 40.29 | Aldeia das Dez, Portugal |  | Sampaio Lab, Portugal |
| ZP614 | 48.98 | Murrhardt, Germany |  | Sampaio Lab, Portugal |
| ZP1167 | 42.57 | Cantabria, Spain |  | Sampaio Lab, Portugal |
|  |  |  |  |  |
| FAR EAST |  |  |  |  |
| CBS8436 | 43.12 | Vladivostock, Russia |  | this study |
| CBS8437 | 43.12 | Vladivostock, Russia |  | this study |
| CBS8439 | 43.24 | Ternei City, Russia |  | this study |
| CBS8440 | 41.30 | Sikote-Alinsky reserve, Russia |  | this study |
| CBS8441 | 41.30 | Sikote-Alinsky reserve, Russia |  | this study |
| CBS8442 |  | Khazansky District, Russia |  | this study |
| CBS8444 | 43.12 | Vladivostock, Russia |  | this study |
| N_44/CBS8438 | 43.24 | Ternei City, Russia |  | Yue et al. 2017 |
|  |  |  |  |  |
| NORTH AMERICA |  |  |  |  |
| YPS138 |  | Pennsylvania, USA |  | Yue et al. 2017 |

* Strains Yxx, Zxx were isolated in 2003 (Koufopanou et al. 2006); Qxx, Txx in 1998; Sxx in 1997 (Johnson et al. 2004); ZPxx in 2011.

** Chernobyl area only; strains isolated in 2009.

Appendix II. Genome assembly statistics

|  |  |  |  |  |  |  | Consensus sequence | | | |
| --- | --- | --- | --- | --- | --- | --- | --- | --- | --- | --- |
| Strain/CBS NO. | Sequencing Method | Provider(1) | Initial reads | Mapped reads (2) | % Ref. covered | Mean coverage (3) | Length (bp) | %>Q40 | %>Q30 | % Ns (4) |
| EUROPE |  |  |  |  |  |  |  |  |  |  |
| CBS432 (Ref) | PacBio |  | NA | NA | 100 | 1 | 12010236 | NA | NA | 0.04 |
| CH1 | Illum-HiSeq | GATC | 43511452 | 10288124 | 94.9 | 85 | 11392458 | 99.5 | 100 | 1.03 |
| CH2 | Illum-HiSeq | GATC | 27863022 | 9058222 | 94.8 | 75 | 11380055 | 99.3 | 100 | 1.70 |
| CH4 | Illum-HiSeq | GATC | 17138048 | 8569486 | 94.7 | 71 | 11377095 | 99.5 | 100 | 1.08 |
| CH7 | Illum-HiSeq | GATC | 11073428 | 5569046 | 94.8 | 46 | 11381043 | 99.1 | 100 | 2.86 |
| CH10 | Illum-HiSeq | GATC | 14486338 | 6381498 | 94.8 | 53 | 11381996 | 99.5 | 100 | 1.12 |
| SIG1 | Illum-HiSeq | GATC | 17123366 | 7028894 | 94.9 | 58 | 11395940 | 99.6 | 100 | 0.94 |
| DBVPG4650 | Illum-HiSeq | GATC | 32893878 | 11747658 | 94.8 | 97 | 11384964 | 99.7 | 100 | 0.62 |
| CECT10176 | Illum-HiSeq | GATC | 27097756 | 7982756 | 94.7 | 66 | 11371692 | 99.6 | 100 | 0.64 |
| T76_6 | Illum-HiSeq | GATC | 20586656 | 8601740 | 94.8 | 71 | 11382056 | 99.5 | 100 | 1.20 |
| Z3 | Illum-HiSeq | GATC | 10025838 | 5012346 | 94.7 | 41 | 11379728 | 99.5 | 100 | 1.09 |
| T8_1 | Illum-HiSeq | GATC | 13919884 | 5429578 | 94.8 | 45 | 11383376 | 99.5 | 100 | 1.06 |
| Q59_1 | Illum-HiSeq | SGRP2 | 6127096 | 3729740 | 94.8 | 33 | 11382571 | 0 | 100 | 0.88 |
| Q95.3 | Illum-HiSeq | SGRP2 | 3959230 | 2605524 | 94.7 | 23 | 11378179 | 0 | 100 | 1.49 |
| S36.7 | Illum-HiSeq | SGRP2 | 4767274 | 2714140 | 94.8 | 24 | 11380037 | 0 | 100 | 1.44 |
| Y7 | Illum-HiSeq | SGRP2 | 4395732 | 2707793 | 94.7 | 24 | 11377555 | 0 | 100 | 1.56 |
| Y8_5 | Illum-HiSeq | SGRP2 | 61016938 | 43048246 | 95 | 349 | 11409081 | 99.7 | 100 | 0.31 |
| Y9_6 | Illum-HiSeq | SGRP2 | 49619328 | 14672254 | 95 | 133 | 11407157 | 99.5 | 100 | 0.52 |
| Z1 | Illum-HiSeq | SGRP2 | 44646838 | 10270718 | 94.9 | 94 | 11403824 | 99.3 | 100 | 0.64 |
| Z1_1 | Illum-HiSeq | SGRP2 | 51505568 | 33307692 | 95 | 271 | 11406393 | 99.7 | 100 | 0.30 |
| ZP600 | Illum-MiSeq | IGC | 6348190 | 4285762 | 95.8 | 107 | 11501331 | 0 | 100 | 1.26 |
| ZP614 | Illum-MiSeq | IGC | 2952972 | 2220167 | 95.9 | 53 | 11513922 | 0 | 100 | 1.58 |
| ZP1167 | Illum-MiSeq | IGC | 4460228 | 3105242 | 95.5 | 63 | 11476189 | 3.5 | 100 | 0.67 |
|  |  |  |  |  |  |  |  |  |  |  |
| FAR EAST |  |  |  |  |  |  |  |  |  |  |
| N_44/CBS8438 (Ref) | PacBio |  | NA | NA | 100 | 1 | 11813124 | NA | NA | 0.03 |
| CBS8436 | Illum-HiSeq | GATC | 16934296 | 6855668 | 96.7 | 57 | 11420815 | 99.4 | 100 | 1.41 |
| CBS8437 | Illum-HiSeq | GATC | 17785536 | 7563016 | 96.7 | 63 | 11418862 | 99.5 | 100 | 1.05 |
| CBS8439 | Illum-HiSeq | GATC | 19314452 | 10307260 | 96.8 | 87 | 11434157 | 99.7 | 100 | 0.57 |
| CBS8440 | Illum-HiSeq | GATC | 15109470 | 6953550 | 96.7 | 58 | 11425297 | 99.6 | 100 | 0.77 |
| CBS8441 | Illum-HiSeq | GATC | 27034090 | 6594710 | 96.7 | 55 | 11425451 | 99.6 | 100 | 0.80 |
| CBS8442 | Illum-HiSeq | GATC | 11696290 | 6372380 | 96.7 | 53 | 11418285 | 99.6 | 100 | 0.88 |
| CBS8444 | Illum-HiSeq | GATC | 17264520 | 9754986 | 96.8 | 82 | 11433877 | 99.7 | 100 | 0.55 |
|  |  |  |  |  |  |  |  |  |  |  |
| NORTH AMERICA | |  |  |  |  |  |  |  |  |  |
| YPS138 (Ref) | PacBio |  | NA | NA | 100 | 1 | 11811499 | NA | NA | 0.04 |

(1) Note all sequences were assembled in this study - except for the three reference sequences that were taken from Yue et al. 2017.

(2) (MQ254 Map nearby)

(3) Across reference

(4) Across consensus
